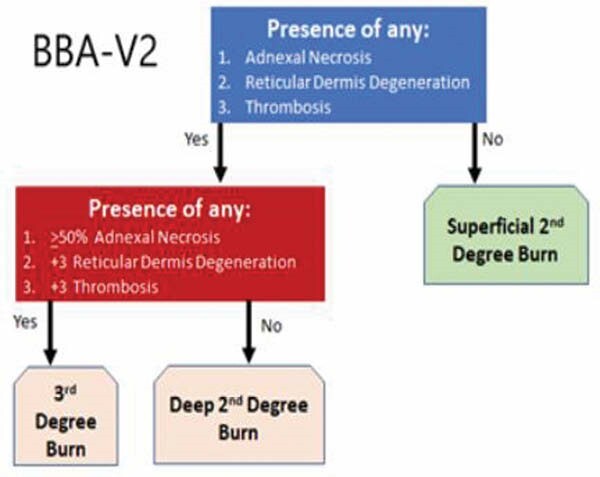# 52 Refinement of a Histologic Algorithm for Burn Depth Categorization Using 1142 Consecutive Burn Wound Biopsies

**DOI:** 10.1093/jbcr/irac012.055

**Published:** 2022-03-23

**Authors:** Herb A Phelan, James H Holmes, William L Hickerson, Clay J Cockerell, Jeffrey W Shupp, J Michael DiMaio, Jeffrey E Carter

**Affiliations:** LSUHSC-New Orleans Department of Surgery, New Orleans, Louisiana; Atrium Health Wake Forest Baptist, Winston-Salem, North Carolina; Spectral MD, Avita Medical, AccessPro Med, Memphis, Tennessee; Cockerell Dermatopathology, Dallas, Texas; MedStar Washington Hospital Center, Washington DC, District of Columbia; Baylor Scott and White, Dallas, Texas; University Medical Center- New Orleans, New Orleans, Louisiana

## Abstract

**Introduction:**

Our group previously reported a theoretical burn biopsy algorithm (BBA-V1) for the categorization of burn wound depth based on histologic analysis, and informed it with the largest series of burn wound biopsies in the literature. That iteration of the BBA resulted in clinical misclassification rates consistent with past literature. Since our last report of that process, we have refined the algorithm with new criteria and a larger repository of burn wound biopsies. Here, we sought to promulgate this newer, simpler version of the BBA (BBA-V2).

**Methods:**

This was an IRB-approved, prospective, multicenter study. Patients with burn wounds assessed by burn experts as requiring excision and autograft underwent 4mm biopsies procured every 25cm^2^. Serial still photos were obtained at enrollment and at excision intraoperatively.

Using H&E with whole slide scanning, a board-certified dermatopathologist assessed each burn biopsy. The criteria used for categorization of burn wound depth in BBA-V1 were: 1) proportion of necrotic adnexal structures, and 2) presence/absence of each of epidermis, papillary dermis, and reticular dermis. The criteria used for BBA-V2 were: 1) magnitude of reticular dermal degeneration, 2) proportion of necrotic adnexal structures, and 3) magnitude of vessel thrombosis.

Biopsy pathology results were correlated with still photos by 3 burn experts for consensus of final burn depth diagnosis. Superficial partial thickness (SPT) wounds were considered to be burn wounds likely to have healed without surgery, while deep partial thickness (DPT) and full thickness (FT) were considered unlikely to heal by 21 days.

**Results:**

The development of BBA V-1 was previously informed by 66 subjects with 117 wounds and 816 biopsies, and resulted in wound categorizations as follows: SPT (20%), DPT (43%), and FT (37%). Therefore, according to BBA-V1, 20% of burn wounds were incorrectly judged as needing excision and grafting by the clinical team. The overall cohort was enlarged to 162 subjects with 294 wounds and 1142 biopsies. The most recent 838 burn wound biopsies were then re-reviewed and re-categorized according to the new BBA-V2 criteria and algorithm. Under BBA-V2, 3% of all burn wound biopsies were categorized as superficial partial thickness, 69% were categorized as deep partial thickness, and 29% were categorized as full thickness.

**Conclusions:**

Our study demonstrates that by adding dermal degeneration severity and vessel thrombosis to our previous criterion of adnexal structure necrosis, BBA-V2 had a much higher rate of concordance with visual clinical assessment for burn wounds clinically judged as needing surgical excision. This study serves as the largest analysis of burn biopsies by modern day burn experts.